# A Review of Brain-Computer Interface Games and an Opinion Survey from Researchers, Developers and Users

**DOI:** 10.3390/s140814601

**Published:** 2014-08-11

**Authors:** Minkyu Ahn, Mijin Lee, Jinyoung Choi, Sung Chan Jun

**Affiliations:** School of Information and Communications, Gwangju Institute of Science and Technology, Gwangju 500712, Korea; E-Mails: minkyuahn@gmail.com (M.A.); mijinlee@gist.ac.kr (M.L.); jinyoungchoi@gist.ac.kr (J.C.)

**Keywords:** brain-computer interface (BCI), game, entertainment, opinion survey, market

## Abstract

In recent years, research on Brain-Computer Interface (BCI) technology for healthy users has attracted considerable interest, and BCI games are especially popular. This study reviews the current status of, and describes future directions, in the field of BCI games. To this end, we conducted a literature search and found that BCI control paradigms using electroencephalographic signals (motor imagery, P300, steady state visual evoked potential and passive approach reading mental state) have been the primary focus of research. We also conducted a survey of nearly three hundred participants that included researchers, game developers and users around the world. From this survey, we found that all three groups (researchers, developers and users) agreed on the significant influence and applicability of BCI and BCI games, and they all selected prostheses, rehabilitation and games as the most promising BCI applications. User and developer groups tended to give low priority to passive BCI and the whole head sensor array. Developers gave higher priorities to “the easiness of playing” and the “development platform” as important elements for BCI games and the market. Based on our assessment, we discuss the critical point at which BCI games will be able to progress from their current stage to widespread marketing to consumers. In conclusion, we propose three critical elements important for expansion of the BCI game market: standards, gameplay and appropriate integration.

## 1. Introduction

Brain-Computer Interface (BCI) technology is a rapidly growing field and publicity about this technology is increasing. Although the original aim of BCI technology was to provide a new pathway of communication for patients suffering from complete paralysis, some researchers have focused recently on the application of BCI to games for use by healthy people. Studies have demonstrated examples of BCI applications in such well-known games as “Pacman” [1], “Pinball” [2], “Tetris” [3], and “World of Warcraft” (“WoW”, [4]), as well as new customized games, such as “MindBalance” [5], “Bacteria Hunt” [6], and others [7–12].

According to reports [13], video games already occupy a large scale market. BCI games may be a very promising area for application of this technology in the near future. Allison *et al.* [14] mentioned the gamer as the first user of BCI technology if BCIs provide useful functionality. In addition, van Erp and his colleagues have predicted that the first mass application of nonmedical BCIs will be in gaming and entertainment [15]. The fBNCI (www.future-bnci.org) project announced that BCI for gaming is second among the top five most promising applications after BCI for assistive technology [16]. Among BCI applications, the number of potential users in BCI games is highest [16,17] and its economic viability is also high [15]. In addition, convenient electroencephalography (EEG) devices equipped with electrodes that do not require gel have been released on the market [18]. Considering these opinions about the positive perceptions of BCI games and the development of EEG devices for the public, it seems clear that BCI games will soon be the first popular application of the technology, although the clinical application of BCI is frequently addressed as futuristic promising application.

However, compared to conventional interfaces, most BCI games have been shown to have low performance in terms of accuracy and speed [19]. Further, it has been difficult to find a successful commercial product using current research approaches. This means that there may be some discrepancy between research and development. Actually, researchers might have little experience in game development. Most likely, they focus on how to integrate BCI with a game rather than on game content. Therefore, studies usually focus on testing system performance or showing the feasibility of BCI. This is likely to lead to a BCI game that is reliable, but less interesting to the user. On the other hand, companies and users will be attracted to games that are entertaining and exciting. In this sense, Gürkök *et al.* [20] suggested two descriptors that both researchers and developers need to consider before designing BCI games. One is game playing motivation, which specifies challenge, fantasy and sociality as key factors. Gürkök [21] pointed out that people play games that may fulfill their psychological needs, and they perceive a correspondence between certain psychological needs and game playing motivations: competence, challenge, relatedness and socialization. Such positive motivations lead to a game that is satisfying to the user because it provides enjoyable content. The second descriptor is the interaction paradigm (action and reaction of the user to events or feedback given in the BCI application) on which the game is built. The latter is important, because most BCI research is conducted with a limited number of control paradigms, each with its own problems or limitations. This should be the focus of game designers until researchers develop a new paradigm that is free from the limitations of existing control paradigms.

In Marshall's recent study [21], BCI games and specified properties were reviewed according to the game genre, such as action, strategy, role-playing, adventure, sports, simulations and puzzle games; “gameplay” was indicated as the key aspect to be considered in game development. Further, several recommendations were made for genre-specific BCI game development, and assessment of user performance by BCI game developers. This is a valuable step in reducing the gap in knowledge between research and game development, as specifying which genres work well with a specific BCI will allow developers to produce a well-designed game. These studies encourage communication between developers and researchers. However, there is one more group that must be engaged in this interaction. Van Erp *et al.* [15] argued that developers (in some sense, the researcher is in the position of the developer) should involve users in game development as early as possible in order to obtain their systematic feedback.

However, investigations of inter-group interactions have not yet been conducted. A few studies have been carried out to collect different perspectives and opinions of researchers with respect to various issues in BCI [22]. The personal opinions expressed in [22] indicate that most BCI researchers likely come from an engineering background and may have less training and interest in specific issues than do other stakeholders. Thus, BCI researchers' focus may be far different from gamers' or may be less interesting, convenient or necessary to the latter. For example, motor imagery is the paradigm most widely incorporated in BCI research; however, gamers may not accept this paradigm for interaction with a computer. Rather than a complicated system, what the game developer needs may be an automated module that is easy to use and moderately reliable. In the Asilomar survey, Nijboer *et al.* [22] clarified an ethical issue that has rarely been considered by BCI researchers. This type of study is beneficial, in that it provides feedback from one group to another and makes certain groups (for example, researchers, developers and companies) aware of issues that they have not confronted to date, but may in future. The development of BCI games is a matter related not only to BCI researchers, but also to game developers and even gamers. Thus, the voice of those groups is important in the implementation of user-attractive games. For these reasons, we believe that it is valuable to determine the factors that will enable BCI games to enter the market. To do so, it is necessary to understand the public's awareness of BCI and its applications, and to evaluate current BCI methodologies that researchers have developed, as well as the marketability of BCI games [23]. Further, we believe that researchers and developers have their own views and strategies for commercializing BCI games and enhancing the BCI game market. This reciprocal feedback is expected to help us understand the current position and difficulties that each group has faced, are facing, and will face.

To summarize, communication among the various stakeholders in BCI games is crucial now that BCI has become interconnected with games. We believe that this study is timely in understanding the opinions of these groups or at least in emphasizing the necessity of exchanging opinions and feedback. Motivated by the need to gather opinions and concerns from these groups, we provide here the current state of the art in BCI games, and gather opinions about BCI games from different stakeholders. Further, we discuss promising steps in the advent of BCI games. For this purpose, we conducted an extensive literature search survey, administered questionnaires to different groups and gave game demonstrations in order to obtain feedback from users before and after playing games.

In Section 2, the fundamentals of BCI and BCI games are introduced briefly, and the current state of BCI games is presented from the results of our literature search. Section 3 describes the survey procedures and methods that were designed to address the questions that must be answered in order for BCI games to become popular with the public. In Section 4, the survey results are presented and interpreted, in order to assess the public's awareness of BCI's usefulness, what research control paradigms are appropriate for BCI games, opinions on a reasonable price, and whether people believe that BCI is available now or how many years will be needed to develop BCI if they do not believe it is already on the market. Further, through the intensive questionnaire we administered to 294 participants, we investigated the elements that researchers and game developers believe are important in the development of BCI games. Finally, further interpretations and an in-depth discussion are presented in Section 5.

## 2. Review of BCI Games

### 2.1. Closed Loop and Components of BCI

**Figure 1.**
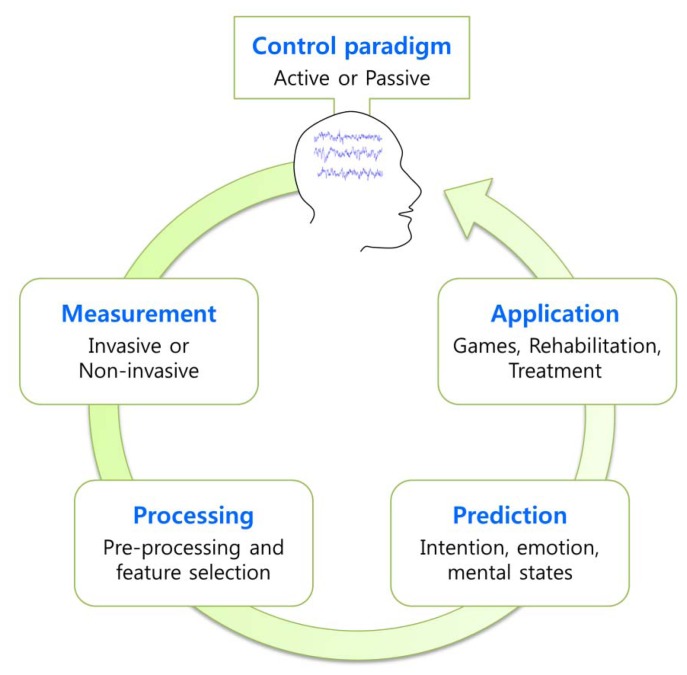
The Closed loop of BCI.

BCI forms a closed loop on its framework, and includes five elements, as presented in Figure 1. These are “Control paradigm”, “Measurement”, “Processing”, “Prediction” and “Application”. Through these five steps, the BCI interprets a user's intention or mental state and uses the information to run the application. Until termination of the system, this closed loop is repeated between the user and the application, with the four modules forming an interface between them. The details of the BCI elements are as follows:

*Control paradigm*: To convey information to the system, a user may push a button according to its function, or in conventional interfaces, move a mouse. However, BCI requires a “Control paradigm” for which the user is responsible. For example, the user may imagine body part movement or concentrate on a certain object to generate brain signals that include the user's intention. In some BCI systems, the user's intentional efforts may not be required; instead, the system detects the user's mental or emotional states automatically. From an interactive viewpoint, these are categorized as active, reactive or passive approaches [24]. The details of control paradigms will be addressed in Section 2.2.
*Measurement*: Brain signals can be measured invasively or non-invasively [25]. Invasive methods, such as electrocorticogram (ECoG), single micro-electrode (ME) or micro-electrode arrays (MEA) detect signals on or even inside the brain, thereby guaranteeing relatively good quality signal. However, these procedures necessitate surgery and the risks associated with it; thus, invasive approaches are clearly unsuitable for healthy people. Therefore, considerable BCI research has been conducted through non-invasive measurements [25], such as EEG, magnetoencephalography (MEG), functional magnetic resonance imaging (fMRI) and Near-Infrared Spectroscopy (NIRS), among others. Of these methods, EEG is the most popular [26]. Compared to other measuring devices, EEG is cheap and portable; further, wireless EEG devices are now available on the market at reasonable prices. Therefore, EEG is the most preferred and promising form of measurement method for application to BCI games.
*Processing*: The brain signals measured are processed to maximize the signal-to-noise ratio and select target features. In this step, various algorithms [27,28] are applied, such as spectral and spatial filtering, to reduce artifacts as well as to extract informative features. The selected target features are used as inputs for classification or regression modules.
*Prediction*: This step makes a decision regarding the user's intention or quantifies the user's level of emotion and mental states. For prediction, classifiers such as threshold, linear discriminant analysis, support vector machine, or artificial neural network are usually employed. For an extensive survey on the algorithms related to processing and prediction, please refer to the literature [27,29].
*Application*: Once the user's intention is determined in the prediction step, the output is employed to change the environment of the application: for example, games [19]; rehabilitation [30], or treatment systems for attention deficit hyperactivity disorder [31]. Finally, the change in the application is delivered to the user as feedback.

### 2.2. Examples of BCI Games

In this section, we introduce the three types of BCI in the context of interaction between a human and a computer, adopting the definition in the literature [24,32]. Typical examples of existing games will be given in order to help the reader understand them. With respect to the categories of control paradigm, we will discuss active, passive and reactive BCI games. For more extensive BCI game reviews, refer to [19,33].

#### 2.2.1. Active BCI

Active BCI is a commonly used paradigm that requires a user to generate brain signals actively; in order to be used for control, these should be discriminative. Active BCI can be adopted for direct control of an application. In one study [2], a pinball game employed BCI for control of the left/right paddles. The system was designed to discriminate two classes of motor imagery, such as left or right hand movement. Motor imagery is the paradigm used most often in BCI research [26]. The key feature between these two thought patterns is contra-lateral event related de-synchronization (ERD), along with ipsi-lateral event related synchronization (ERS; [34]). When a user imagines right hand movement, the amplitude of activity in the mu-rhythm in the sensory-motor area of the brain decreases in the left hemisphere and increases in the right hemisphere. For motor imagery of the left hand, the converse occurs. Therefore, the motor imagery yields spatially different brain activity according to which hand is employed. Through signal processing and classifiers, BCI can detect spatially different patterns and decide which direction the user intends. Then this direction information is used to control paddles (left or right paddles). This is the same as conventional interfaces, such as left or right arrows on a keyboard. As its name implies, the user initiates brain activity; thus, the information embedded in the signal is captured and employed to control the movement of the paddles.

#### 2.2.2. Reactive BCI

While a user generates an informative brain signal consciously in active BCI, this is not always the case. In reactive BCI, information with respect to the user's intention is embedded in the response signal to stimulation. The user can cause his/her brain signals to maintain the intended information simply by attending to or looking at certain stimuli. For example, the “MindBalance” game [5] uses the steady state visual evoked potential (SSVEP). Checkerboards flickering at 17 and 20 Hz are placed on the left and right sides of an avatar. To maintain the avatar's balance, the system requires the user to focus on either the left or right checkerboard. In response to the visual stimulation, the brain activity in the occipital area includes the correlated frequency information. For example, the brain signal oscillates at 17 Hz if the user attends to the checkerboard flickering at 17 Hz. Through this mechanism, the BCI system knows at which checkerboard the user is looking, which enables the user to balance the avatar. P300 paradigm, as an example of SSVEP, is also widely used in developing reactive BCI. P300 component is the positive deflection that appears roughly 300 ms after stimulus presentation. The amplitude and latency of this component are related to attention of the subject to the stimulus, which may be applied to spellers [35].

#### 2.2.3. Passive BCI

In passive BCI, the primary role of the system is not to give control to the user, and it does not require any effort on the part of the user; instead, it monitors the user's mental states automatically. It quantifies the level of attention or differentiates emotional states to facilitate interaction between the system and user. For example, the ratio of frequency band powers, such as sensory-motor rhythm (12–15 Hz), beta (13–30 Hz) and theta (4–8 Hz) [8] are used for attention index quantification; for relaxation level [4], alpha (8–13 Hz) power is used. In [8], the system quantified the concentration level, and applied it to adjust the velocity of a car and produce sound effects that gave feedback to the user.

### 2.3. State of the Art in BCI Games

To understand the current status of BCI games, we conducted a literature search using SCOPUS (www.scopus.com), the abstract and citation database of peer-reviewed literature. In order to survey articles related to BCI games, we searched publications from 2007 to 2013 for the following keywords and operators: ((“brain computer interface” OR “BCI”) AND (“game” OR “entertainment”)) in “Article Title, Abstract, Keywords.” Initially, 272 articles were found; this list included conference proceedings, as well as research, review and survey papers. Among these, we excluded articles that were not related directly to BCI games, and brief conference versions of extended journal papers. From this search, we found that approximately half of the application and research papers were published in conference proceedings, but not in journals. Finally, a total of 180 articles was retrieved and used to review the current progress of BCI games.

#### 2.3.1. Publications

**Figure 2.**
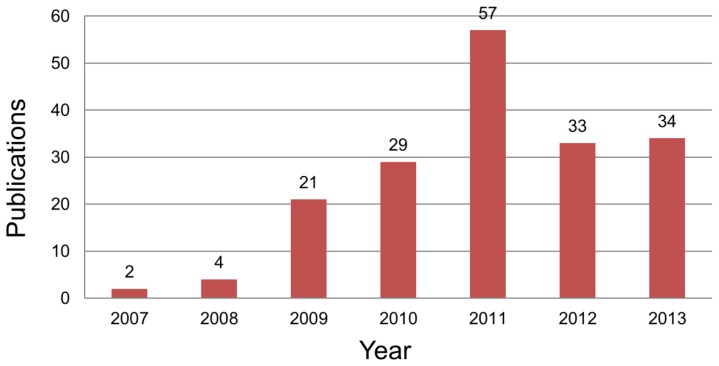
The number of publications over seven years (2007–2013). The results include conference papers, but exclude articles not related directly to BCI games. The search was conducted through October 2013.

Figure 2 presents the number of publications on BCI applications. From 2007 to 2012, the number of publications increased greatly. While a few articles were published during 2007–2008, over thirty articles per year have been published since 2010. The increase in publications peaked in 2011 and stabilized thereafter. We believe that the increase in research may have been influenced by the development of wireless EEG devices. For example, “Neurosky Mindset” (www.neurosky.com) and “Emotiv EPOC” (www.emotiv.com) were released in 2007 and 2009, each at a price more modest than that of other research purpose devices.

#### 2.3.2. Game Development and Application

**Figure 3.**
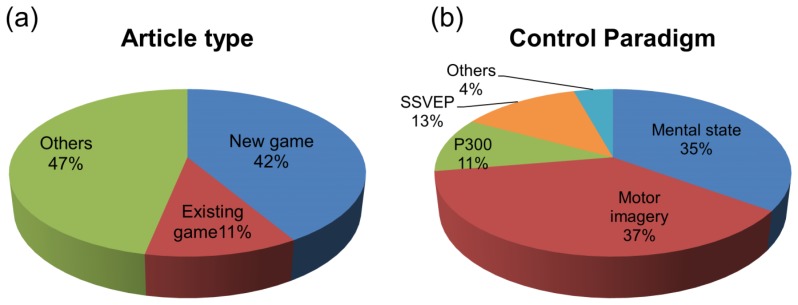
(**a**) All article types (N = 180) and (**b**) control paradigms. Articles were categorized according to whether or not they implemented games and whether the game is an existing or new one. Among articles (N = 92) associated with game development, we investigated the control paradigm employed (here all control paradigms used in a hybrid approach are counted). “Others” indicates those studies related to EMG-based methods.

Although we found 180 published articles, not all of them were directly related to the implementation of BCI games. Therefore, we separated studies on the development of BCI games from others, such as review papers, in order to help us understand how many games were actually developed during this trend of increased publications on BCI games. We found that approximately 47% of publications did not develop games, while the remaining studies did: newly-developed games (42%), or BCI implementation with existing games (11%), such as Tetris, Hangman, and World of Warcraft, are shown in Figure 3a. Excluding articles unrelated to game development, we categorized those remaining according to the control paradigm used; only half applied BCI to games, regardless of the interface used for the purpose of control or interaction between the game and user. Consequently, with which paradigm(s) and for what purpose BCI is used are interesting questions. Among the studies that implemented BCI games, we inspected the control paradigms that were employed. In articles associated with a hybrid approach (for example, P300 and motor imagery), all control paradigms used were counted. In Figure 3b, the majority of studies (61%) used active (motor imagery: 37%) or reactive (P300: 11%; SSVEP: 13%) BCIs, while 35% of the studies used passive BCI (mental state), such as quantifying attention, emotion and fatigue. A small percentage of the studies were related to electromyography (EMG)-based methods, such as eye-blinking.

#### 2.3.3. Hardware

**Figure 4.**
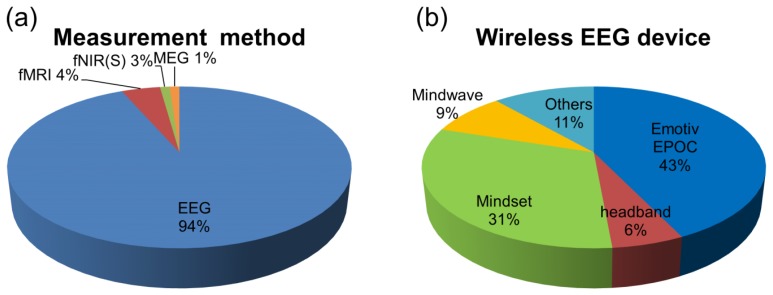
(**a**) Measurement methods (N = 92) and (**b**) wireless devices (N = 35) used in developing BCI games.

The chart in Figure 4a shows the measurement methods that have been introduced in developing BCI games (N = 92; this information was identified clearly in 92 articles.). One can see readily that EEG is a well-used measurement method in research that incorporates games. In this context, observing which device is used for BCI game development is an important aspect in understanding current trends. The chart in Figure 4b shows the use of wireless EEG devices in studies related to BCI game development. Notable devices are “MindSet” and “Emotiv EPOC.” It is likely that the preference for “Emotiv EPOC” is due to the fact that it has 14 channels, more than any other. For example, “MindSet” has one channel and others have ten channels at most. Moreover, the location of channels in “EPOC” includes centro-occipital areas. These advantages broaden the use of various control paradigms, such as motor imagery, P300 and SSVEP.

The dramatic increase in publications after 2007 (Figure 2) seems to have been caused by the release of wireless EEG devices. Because of their ease of use and low cost, we believe that as the market in EEG devices matures, more researchers will choose EEG as measurement method. Table 1 shows the EEG devices currently on the market or in preparation for marketing. Briefly, nine devices are notable, most of which support wireless communication with reasonable sampling rates from 128 Hz to 1 kHz. Considering that most BCI research extracts features below 100 Hz [17], the sampling rate is sufficient to implement BCI. Except for “Emotiv EPOC”, none of these devices supports electrodes covering the central area; instead, most of them measure brain waves on the forehead. In our web search, we found three products—“Melon EEG headband”, “InteraXon Muse” and “Emotiv Insight”—that are gaining public attention by reaching their target figure through the funding platform “Kickstarter” (www.kickstarter.com). Looking at the success of this fundraising effort, “Emotiv Insight”, for example, achieved over 1600% of its goal; this fact demonstrates the strong interest of the public in BCI and BCI hardware.

## 3. Methods

### 3.1. Survey Contents

Our goal was to understand the discrepancies and similarities in opinions, and to bridge the gap among various stakeholders in the area of BCI games—producers (researchers and developers) and consumers (users). In this survey, we confined our inquiry to researchers working in the BCI or bio-signal processing fields, and regarded respondents who are neither researchers nor game developers as those who may use BCI games for entertainment. For simplicity, we referred to respondents in these three groups as researchers, developers and users.

The survey was designed to obtain personal opinions about the applicability of BCI or BCI games, how usable or acceptable respondents considered the most often used control paradigms in the BCI field, and expectations/considerations that BCI games will become common entertainment in our daily lives. The questionnaire procedure was as follows. At the beginning of the survey, a consent form was given to and signed by the participants. In step 2, personal information was gathered and then brief explanations about BCI were presented with pictorial examples. After this initial process, the main survey began, but the form of the questionnaire was designed slightly differently depending on the group to which the respondent belonged. For researchers and developers, we asked the number of years they had spent in their career and what they thought were the important elements for BCI games and for stimulating the field of BCI game development. Users were not asked these questions; instead, users/developers were asked whether/how they knew about BCI. Apart from these, most of the questions were the same. There were 10 to 12 items depending upon the group. Questions common to all groups are as follows.

*The usability of BCI*: Awareness of the applicability and influence of BCI and BCI games is an interesting issue. To obtain opinions with respect to those issues, we first asked respondents to express their evaluations of the future applicability and influence of BCI and BCI games. Second, several examples were presented and respondents were asked to state their personal opinions of the applicability for each purpose. In these questions, respondents' opinions were recorded on a five-point Likert scale as “very high”, “high”, “not strong”, “low” and “very low”. For example, we asked the following question: “Please state your evaluation of the applicability and influence of BCI in the future”. One of five opinions was received from each participant.
*The preference rating for BCI paradigms*: The overall system environments, such as the task that a user has to perform, the sensor location and the stimulation may all depend on the control paradigm. Therefore, evaluating the control paradigm is an important issue, as we do not know whether users will readily accept the system environments of BCIs that are now considered promising. As we stated in Section 2, we described three types of control paradigms that are used most widely, especially in non-invasive BCI. With active, reactive and passive BCI control paradigms, we specified their functionality in the context of interaction and asked users and developers to give their personal evaluations of the paradigms on a five-point Likert scale ranging from “not interesting”, “somewhat interesting”, “fairly interesting” and “very interesting” to “one of the most interesting”. For this question, we introduced motor imagery as active BCI, P300 and SSVEP as reactive BCI, and mental state as passive BCI. This question was presented more directly to the researchers, who were asked about their interest in active, reactive and passive BCI.
*Sensor position*: With respect to the control paradigms, we may distinguish three important brain areas; frontal, centro-parietal and occipital. In addition to these, we can also use the overall area covering from frontal to occipital. In this question, the respondents were given four sensor locations, and instructed to order them according to their preference. To help the respondents understand the question, it was presented with illustrations of positions on the head.
*Reasonable costs*: For guidelines pertaining to cost, we provided the reference cost of two recognizable devices, Microsoft “Xbox Kinect” (www.xbox.com, $300∼500) and Nintendo “Wii” (www.nintendo.com/wiiu, $300). We thought that it would be helpful for the respondents to understand the trends in price of the new interface devices, although this is not absolutely comparable information. Thus, we set up the items as, “less than $100,” “between $100 and $200,” “between $200 and $300,” “between $300 and $400,” and “over $400.”
*Expected years until availability*: We also asked the respondents how many years they thought it would be before such games would be available to the public. This question included 6 items specifying a range of years.

### 3.2. Pilot Study

A pilot study was conducted with 20 respondents who are BCI researchers currently working at our institute, as well as game developers and members of the public in Korea. The form was distributed to them and they were asked to complete the questionnaires and give feedback with respect to the completeness or ambiguity of sentences in the questions, any possible difficulties in conducting the survey, and further comments. Through this procedure, we found that completion of the survey took approximately 5 min; we were also able to correct and revise the survey items to ensure that their meanings were conveyed in a clear and unambiguous manner. In addition, we added one more example in the question for “BCI paradigm”: “speeding up the velocity of a car through the user's concentration level” [8], because we found that “changing a character according to user's mental state” [4] might not be a sufficiently clear example of passive BCI. Finally, the updated version was released to the public through the internet.

### 3.3. Final Survey

The final form of the survey is presented in the appendix. Survey participants were recruited through various channels. We posted promotional articles on social network services, such as Facebook, Twitter, LinkedIn, BCI News (www.neurogadget.com), and other community bulletin boards. In addition, we notified societies and community groups about the survey (e.g., BCI2000, neurogaming conference, American Psychological Association, Society for Game Developers in Korea, and International Game Developers Association). The survey was conducted between August and October 2013.

### 3.4. Statistical Tests

In order to determine which opinions among the groups were statistically significant, we applied the Wilcoxon rank-sum test with the hypothesis that the medians of two variables (each variable represents one group opinion for the given question) are equal. For this statistical test, answers to the given question were properly digitized (transformed into integers)–for example, “one of the most interesting” was assigned a 5, “very interesting” a 4, “fairly interesting” a 3, “somewhat interesting” a 2, and “not interesting” a 1.

## 4. Results

### 4.1. Respondent Profiles

A total of 294 people participated in the survey. The details of respondents' profiles are presented in Table 2. Five profiling questions indicated how many respondents belonged to each sub-group, using percentages of all participants (N = 294). There were 208 male and 86 female respondents. The majority of respondents (183: 62%) was between 20 and 29 years of age and 71 (24%) were between 30 and 39 years. Over half of the respondents live in Asia, with 22% in North America, 18% in Europe, 4% in South America, and approximately 1% or less in Australia and in Africa. In the question referring to hours of game-playing, 95 respondents (32%) answered that they spent less than 30 min per day playing games, while 57 respondents (19%) did not play games. Nearly the same number of respondents answered that they played games between 30 min to 1 h (43 respondents: 15%), 1 to 2 h (50 respondents: 17%) and more than 2 h (49 respondents: 17%). Overall, there were 90 researchers (31%) and 36 (12%) developers among the 294 respondents. 168 (57%) respondents were users (neither researchers nor developers) and thus, potential gamers. Please note that, throughout this study, results are presented as percentages by groups (users, researchers, developers). Within each group, we found that the researchers' group had the highest mean age (users: 27.6 ± 7.4; developers: 28.6 ± 6.7; researchers: 30.5 ± 7.4) and they tended to spend less time playing games than others (researchers: 28.3 ± 33.1 min; users: 50.1 ± 46.3 min; developers: 77.1 ± 38.3 min).

**Figure 5.**
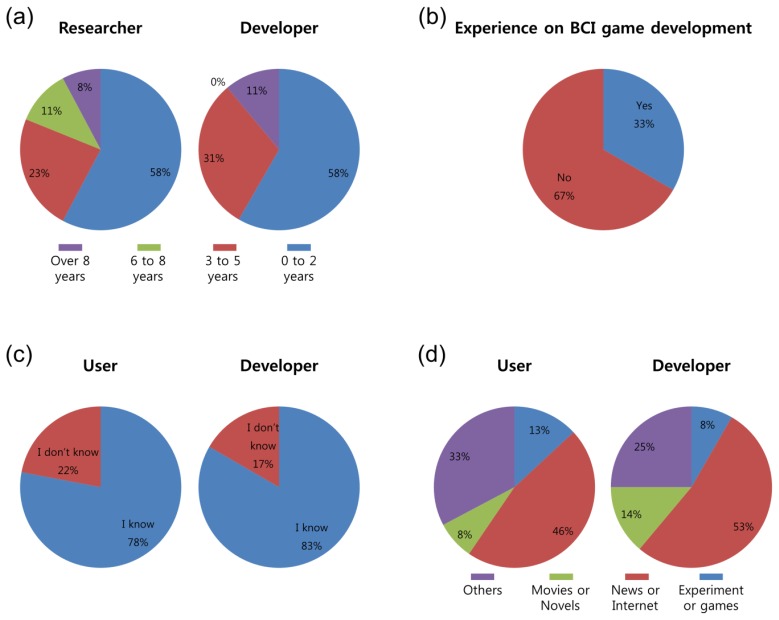
BCI and BCI game background. Responses to the questions: (**a**) years in research or development; (**b**) experience with the development of BCI games for researcher-only question; (**c**) whether the respondent knew of BCI or not; (**d**) how they knew about BCI. The percentages were calculated as percentages of users (N = 168), developers (N = 36) and researchers (N = 90).

In Figure 5, the backgrounds of respondents are presented as percentages of the total number of respondents. First, the length of the researchers' and developers' careers was not long; as shown in Figure 5a, over half of the researchers and developers answered that they had spent less than 2 years in their field. For researchers, we asked whether s/he had developed any BCI games. Approximately 33% of the researchers responded that they had experience in the development of BCI games. 78% of users and 83% of developers already knew what BCI was, while only a small fraction of respondents were unaware of BCI. Participants stated that they had heard of BCI technology in the news or on the internet (users: 46%; developers: 53%), in movies or novels (users: 8%; developers: 14%), by participating in experiments or games (users: 13%; developers: 8%) and by other means (users: 13%; developers: 8%). When asked specifically about other means, the respondents stated that they had heard of BCI from their friends or from their own studies.

### 4.2. Applicability and Expectations

**Figure 6.**
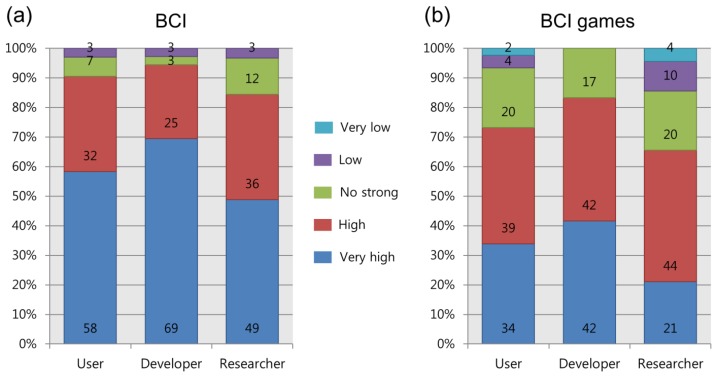
Respondents' evaluations of the influence of BCI (**a**) and BCI games (**b**). All three groups answered that BCI and BCI games would have a considerable influence in the future. The percentages were calculated by groups.

All three groups were asked about their awareness of the applicability and influence of BCI and BCI games. As shown in Figure 6, we found that all three groups had positive opinions with respect to the future influence of BCI and BCI games. For BCI technology, 90% (users), 94% (developers) and 85% (researchers) of respondents responded “very high” or “high” with regard to the influence of BCI, while only 3% in each group answered “low” or “very low”. In the question about BCI games, the positive opinions decreased somewhat. 65% of researchers, 73% of users, and 84% of developers responded that BCI games would have a “high” or “very high” influence.

**Figure 7.**
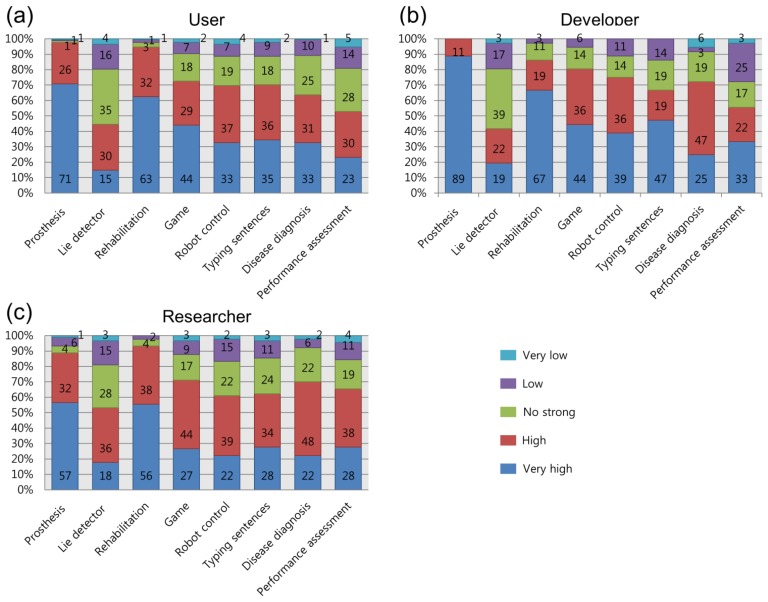
Respondents' evaluations of the applicability of BCI in specific areas: users (**a**); developers (**b**) and researchers (**c**).

For the second question, respondents indicated on the same scale their perceptions of the influence of each application example presented. Overall, the respondents agreed that BCI applications had high potential, but the degree of their opinions varied. These results are presented in Figure 7. The potential of lie detectors did not receive strong agreement, as less than 50% of respondents in each group stated “very high” or “high”, while over 50% of respondents agreed on their potential in other applications. Many of the respondents indicated that the application to rehabilitation systems and prostheses would have high potential in the future. For rehabilitation systems, 95% of users, 86% of developers and 94% of researchers agreed on its high applicability, while 97% of users, 100% of developers and 89% of researchers also agreed on the high applicability of BCI to prostheses. Interestingly, in the developer group, the majority of opinions were “very high” for rehabilitation systems, but only “high” for prostheses. Apart from these two fields, games seemed to be the next most promising area of BCI application. Many respondents answered “very high” or “high” (users: 73%; developers: 80%; researchers: 71%).

### 4.3. Control Paradigm and Channel Location

**Figure 8.**
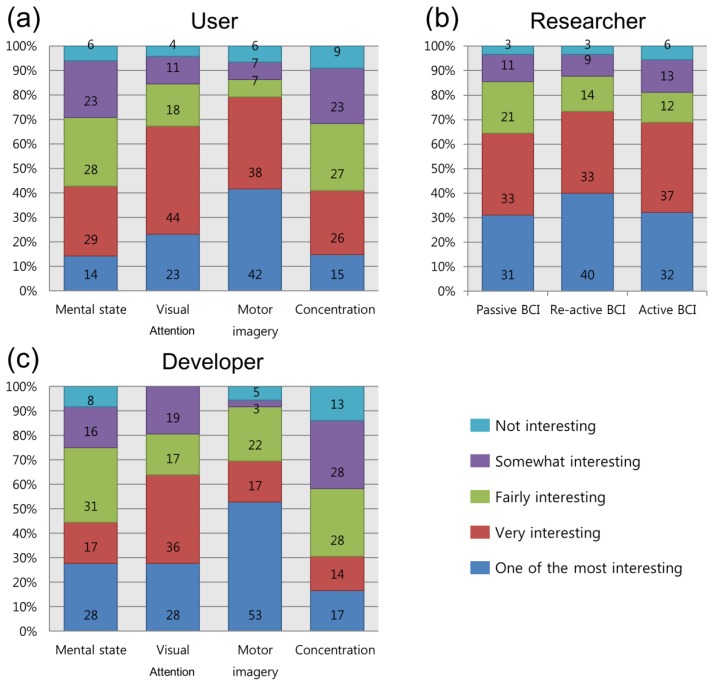
Interest in each paradigm (application): users (**a**); researchers (**b**) and developers (**c**).

The survey also attempted to evaluate the BCI control paradigms. From the results in Figure 8, we observed that a large group believed that motor imagery (users: 80%; developers: 70%) and visual attention (users: 67%; developers: 64%) were “very interesting” or “one of the most interesting.” For passive BCIs, the two applications did not seem to be attractive to users and developers and less than 50% of the respondents in each group answered “very interesting” or “one of the most interesting.” Most of the developers were very interested in motor imagery (“one of the most interesting”: 53%). In the researchers' opinion, reactive BCI was the most interesting of the three types, but the percentages were similar (active: 69%; reactive: 74%; passive: 64%). Another point on which the opinions of users and developers differed from those of researchers is that researchers considered passive BCI (64%) to be as interesting as active and reactive BCI.

**Figure 9.**
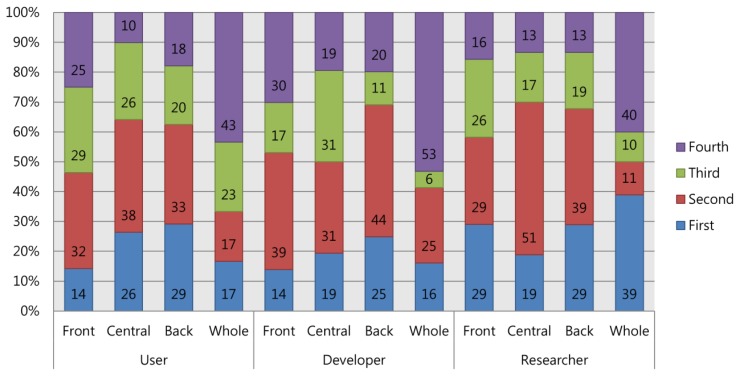
Preference order for sensor positions. A detailed illustration of sensor position is presented in the appendix.

Next, we asked about preferences for sensor positions that may be related to hardware design and thereby may affect the users' convenience. From the results shown in Figure 9, it is clear that it was difficult to determine a distinct preference. However, we attempted to assign a rank to each sensor location in sequence from first to fourth. We found that users' preference order was: “back” >; “central” >; “front” >; “whole”, while developers seemed to prefer the order of: “back” >; “front” >; “central” >; “whole.” Among the researchers, however, we could not find a consistent order; instead, their opinions were divided. Thirty-nine percent of the researchers most preferred “whole,” while the other 40% liked the “whole” sensor position least. From the perspective of users and developers, hardware that covers the whole head is considered to be very inconvenient. This was a clear result, as many of the users (43%) and developers (53%) agreed that they least preferred “whole.” It is worthwhile to provide the statement of one developer who emphasized the importance of design over sensor location: “*The device design is far more important, provided that BCI performance is not heavily depending on sensor location*”.

### 4.4. Price

**Figure 10.**
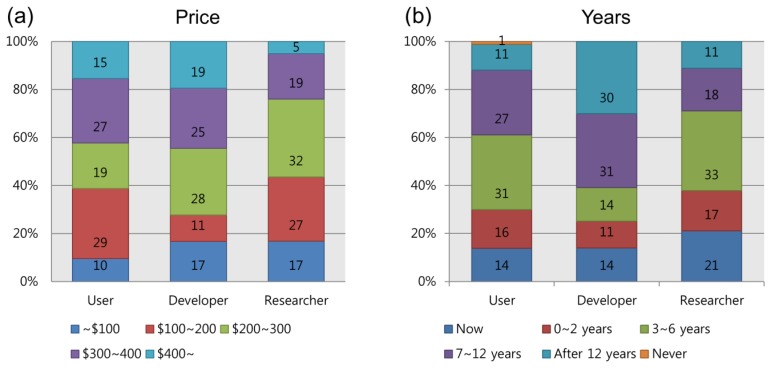
Acceptable cost for BCI games and devices (**a**) and expected number of years until BCI games become generally available (**b**).

Among the elements that influence a consumer's motivation to purchase a product, one of the most important is cost. For this reason, all three groups were asked what they believed was an acceptable price for a device. In Figure 10a, the acceptable price that each group voted for is presented. The most preferable costs were determined as $100∼200 for users, $200∼300 for developers and $200∼300 for researchers. No one preferred the highest priced device, but it is worth noting that more than 80% agreed that a price over $100 was acceptable.

### 4.5. Expected Years until Availability

In a question similar to that of the Asilomar survey [22], we asked users, developers and researchers how many years they expected it would require before BCI games enter the market and become popular. Figure 10b illustrates the responses: (31%) of users expected BCI games to be available within the next 3 to 6 years; 27% answered 7 to 12 years; 16% within 2 years, and 11% over 12 years. 14% of the users believed that BCI games are available already; only 1% predicted that they would never be marketed. Among the developers, the majority expected that marketing would occur either between 7 and 12 years hence (31%), or after 12 years (30%); approximately 14% of respondents answered between 3 and 6 years (14%); within 2 years (11%), and available now (14%). In contrast, the second largest number of researchers (21%) responded that BCI games were available now, although most (33%) expected them to be available from 3 to 6 years in the future. The remainder chose between 7 and 12 years (18%); within 2 years (16%), and over 12 years (11%). Comparing the number of respondents who answered within 6 years, including “now,” we noted that researchers (71%) expected the games would be available sooner than did the other two groups (users: 61%; developers: 39%).

### 4.6. Important Elements

**Figure 11.**
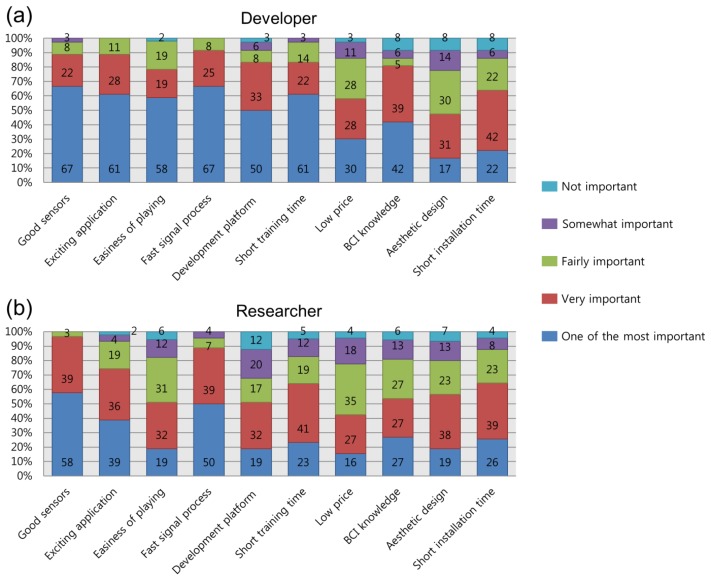
Importance of elements in BCI games: opinions from (**a**) developers and (**b**) researchers.

**Figure 12.**
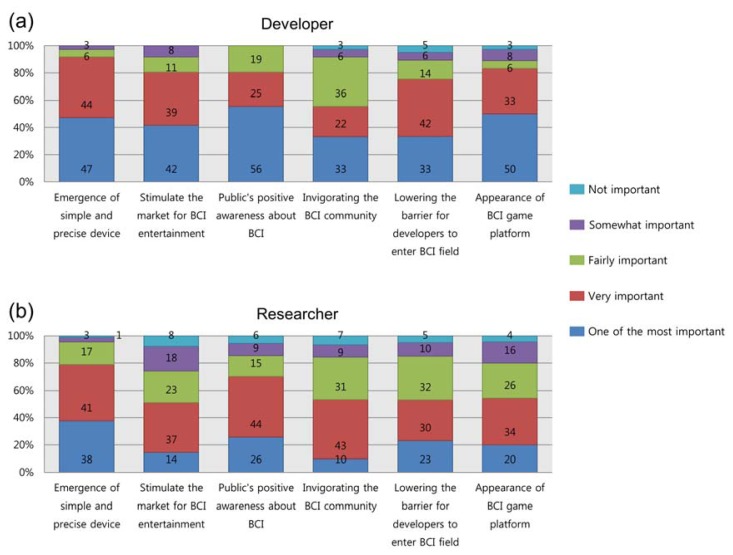
Importance of elements in stimulating the market for BCI games: opinions from (**a**) developers and (**b**) researchers.

We thought that groups that are heavily involved in BCI game implementation might hold different or similar opinions on the subject of the elements important to the public success of BCI games. Thus, every developer and researcher was asked two additional questions. One addressed the importance of the given elements of BCI games, and the second asked how those elements would be important in stimulating the public success of BCI games. They were asked to indicate their agreement with these questions. The results from the two groups are presented in Figures 11 and 12.

As seen in Figure 11, the common elements selected as most important by both developers and researchers were: “good sensors” (developers: 67%; researchers: 58%), and “fast signal processing” (developers: 67%; researchers: 50%). Apart from these, over 40% of developers agreed that “knowledge of BCI” (42%); “short training time” (61%); “platform” (50%); “ease of playing game” (58%), and “exciting application” (61%) are some of the most important elements of games, while 39% of researchers responded that only “exciting application” was the most important element. Developers felt that most elements were more important than did researchers, with the exception of “short installation time”, “device design” and “good sensors”.

With respect to the second question addressed only to developers and researchers, we asked about the importance of the various elements in stimulating the market for BCI games. As we observed in the previous question about the elements required to develop BCI games (Figure 11), developers indicated a higher level of importance for each element in Figure 12 than did the researchers. For most of the elements, 80% of the developers responded either “one of the most important” or “very important,” but not many agreed with the element pertaining to stimulating the BCI community (developers: 55%; researchers: 53%). The two groups agreed that developing communities for interaction was a low priority. With regard to this issue, one researcher said, “*Game developers do not need to study BCI research.*” Similarly, a developer stated, “*Software development kit (SDK) should be provided, so that one can implement games without detailed BCI knowledge*”. From these comments, it is clear that the platform is much more important to game developers. Comparing other elements, researchers tended to pay more attention to two elements: the public's positive awareness (“one of the most important” or “very important”: 70%) and the quality of the device (“one of the most important” or “very important”: 79%), although developers also agreed that these were important.

### 4.7. Statistical Significance of Between-Group Opinions

We conducted a quantitative analysis of our survey and similarities and differences among the opinions of the groups were observed. In addition, a statistical test was conducted as described in Section 3.4. We found that researchers' preference for passive BCI was significantly greater than in the other groups (users and researchers: *p* < 0.0001, developers and researchers: *p* < 0.001), as was mentioned in Section 4.3. For questions related to price and the expected number of years for BCI to reach the market, researchers' opinions of an affordable cost were significantly higher than those of developers (*p* < 0.05) and users (*p* < 0.05). On the other hand, developers expected a significantly longer time would be required for BCI to reach the market than did researchers (*p* < 0.01) and users (*p* < 0.05). With respect to questions about the important elements for a BCI game and for stimulating the market, several elements differed significantly between developers and researchers. For BCI games, “exciting application” (*p* < 0.05); “easiness of playing” (*p* < 0.0001); “development platform” (*p* < 0.0001); “short training time” (*p* < 0.001), and “BCI knowledge” (*p* < 0.05) were significant. For the market, “stimulate the market for BCI entertainment” (*p* < 0.001); “public's positive awareness about BCI” (*p* < 0.01), and “appearance of BCI game platform” (*p* < 0.001) were significant. Overall, these statistical results lend support to the fact that there are different viewpoints among developers, researchers, and users. In-depth discussion on different viewpoints among three groups will be addressed in Sections 5.1 and 5.4.

## 5. Discussion

In this study, we reviewed the current research trends in BCI games and attempted to collect the opinions of all stakeholders: users, developers and researchers. Through our literature search, we observed some notable points. We found that in recent years, a marginal number of articles have shown that there has been continuous research on BCI games, and that motor imagery, P300 and SSVEP-based control paradigms have been introduced widely for control purposes. Moreover, the passive paradigm that reads mental or emotional states accounted for a relatively large percentage of the control paradigms used (35%). We also confirmed that EEG is the most preferred measurement method in the development of BCI games, and we expect that this preference for EEG will continue with the advent of wireless EEG devices. A questionnaire was conducted with nearly 300 participants in order to investigate their awareness of and opinions about BCI games. Here are the points that we learned from the survey:

- BCI was well recognized among users and developers, although only a small fraction of them had experienced this technology. News and the internet were found to be the primary sources of information about BCI.
- All three groups (researchers, developers and users) agreed that both BCI and BCI games would have a large influence as an applied technology in the future.
- Prostheses and rehabilitation were distinguished as the most promising applications, followed by games.
- With respect to the control paradigm, users and developers indicated a preference for active and reactive BCIs, while researchers preferred reactive BCI.
- Users and developers gave the lowest priority to the whole channel covering the head; interestingly, however, the researchers were divided. One group (39%) most preferred the whole head sensor position, while the other (40%) liked it least.
- The majority of each group thought that an affordable price for a BCI device is over $100. Considering that the existing EEG devices or those that are being prepared for the market are priced at approximately $200, the cost to develop BCI generally meets the price acceptable to users.
- The researchers expected that BCI games would be available sooner than did the other two groups.
- As important elements in BCI games, researchers were most concerned with the practical obstacles that they face or will face in their studies, such as signal processing, sensors and ways to make games interesting. On the other hand, developers seemed to take into account the user's view, as well as their own position, more than did the researchers. Compared to researchers, they gave a higher rank to “the easiness of playing” and the “development platform”.
- When asked about elements important in stimulating the BCI game market, developers and researchers agreed that creating communities for interaction was a low priority. However, the developers reiterated the importance of the platform, while the researchers responded that “emergence of simple and precise device[s]” and the “public's positive awareness about BCI” are high priorities.

There is considerable work that remains to be performed before BCI enters smoothly into the field of entertainment. BCI games need to be evaluated continuously by the public by giving them particular experiences with the devices, and the current market status should be included in any considerations. From the following, we discuss certain questions raised by and limitations of our study.

### 5.1. Important Elements for BCI Games and the Market

Among researchers, making a game interesting was not as significant as signal processing. In contrast, developers considered the viewpoint of the implementer and gamer more than did the researchers. Looking at the question from a user's point of view, how easily and rapidly a gamer adapts to a new interface or the way a game is played was very important. A gamer is prone to become bored and stop playing if considerable game training is required. One researcher addressed this issue, stating: “*Generating signals should be intuitive, like motor imagery, or made intuitive by context, like selecting items in a menu with (fast) P300 or SSVEP*.” Thus, the interaction between a user and a game should be as intuitive as possible. On the other hand, one developer made the interesting point that: “*It should guarantee no medical side effect*”. There is a report that SSVEP may entail a higher risk of photic or pattern-induced epileptic seizures [36]. This means that medical side effects need to be added to the list of considerations in developing BCI games, and this should be more important when games incorporate stimulation-based interactions, such as those in reactive BCI.

We observed that researchers are greatly concerned with hardware issues, as many of them agreed that simple and precise devices are one of the most important elements. Good signal quality is essential to decode the user's intention or mental state accurately. In recent decades, most research results come from well-customized laboratory setups having a research-purpose acquisition device. In general, this special device is too heavy and expensive for the public to use with MEG or fNIRS, and even in smaller and cheaper systems, such as EEG, it is very inconvenient for the subject, as well as the researcher, to conduct experiments, as they require gel and numerous lines, and are highly sensitive to noise. Therefore, whether the system works outside the laboratory and how to make it reliable, even in very noisy environments, are major challenges. In this sense, researchers also looked forward to the development of a device that is convenient for experimental applications and has a high signal-noise ratio. From these results, we see that both the same and different opinions exist between these two stakeholders in BCI game production. Not surprisingly, developers tended to hold the views of game developers and users, while researchers addressed the issues most crucial to their research.

### 5.2. Active vs. Passive BCI

It is interesting to investigate the opinion on whether active or passive BCI is more promising for marketing in the present or future. In the survey, we found that active BCI, including reactive BCI, was preferred by users and developers. People may be attracted by the greater control that BCI can provide by comparison to other conventional interfaces, as they may be more familiar with terms like “mind-reading” and “thought control”. However, due to the limitations in the performance of (re)active BCI, companies rarely release products or content based on this paradigm; instead, most applications tend to monitor brain states and use these in the interactions with a user. However, passive BCI is gaining interest among researchers as a practical applied method [24,32,37–40]. Apart from this research trend, it is useful to collect opinions from people who have had experience with each paradigm. Recently, we demonstrated two games to the public at two conferences—one using an active paradigm, and the other a passive paradigm. For details of this demonstration, refer to the appendix. We learned two lessons from this demonstration:

- First, it is difficult to satisfy users when control of the game is limited.
- Second, passive BCI may give the user the feeling that s/he is interacting with the game, thereby making it more entertaining.

Such lessons may result from the discrepancy between the two games used, as their game contents were quite different. Moreover, the game design described just one control paradigm, which may not be representative of all control paradigms. For example, depending on system feedback, users may be aware, consciously or unconsciously, that they are manipulating their mental state. However, the fact that active BCI suffers from low reliability, while passive BCI may provide more control, and thus more entertaining games, are important points, although more study is required with well-designed game and environment parameters. Thus, we expect that passive BCI will be promising and can be used in the important elements of games if users and developers understand the merits that only this type of BCI offers.

There are several advantages of passive BCI [32,41]. First, limited performance is less important than in active BCI. Even with the relatively low performance of the passive paradigm, a game can be played because BCI may not be a main input to the whole system, as in “AlphaWoW” [4], which uses the level of relaxation to change the character of an avatar. In the case in which passive BCI is used as the main input (goal), even low performance would be acceptable, as the true value is not determined and perceptible to the user. The second advantage of passive BCI is that a short response time for immediate feedback is unnecessary and even relatively long response times may be input to the game, because the information is reflected in order to make the game more enjoyable and exciting. Third, passive BCI may provide information on real-time updated physical and mental states of users that conventional interfaces (keyboard, mouse and so on) cannot, as it does not require a user to use conscious effort to interact with the system [24]. With these benefits of passive BCI, we believe that this system will occupy a larger market for some time.

### 5.3. Current State of the Market

In the Asilomar survey [22], 72.4% of researchers (N = 145) indicated that BCI for healthy users was already available or would be within 5 years (e.g., by 2015). However, we observed that only 38% of researchers (N = 90) responded similarly (now available: 21%, and within 2 years—e.g., by 2015: 17%). This inconsistency may be due to several factors. As [22] indicated, researchers may have different standards or do not yet have well-informed opinions because BCI is still a new and developing field. Some researchers only regard control-purpose systems as BCI. Another possible reason is that we included another option: “within 3 to 6 years.” Therefore, we cannot exclude the possibility that some respondents chose that option rather than “within 2 years”. Further, it has been two years since the Asilomar survey [22] was conducted. If the number of respondents who chose “within 3 to 6 years” is included, then the percentage is 71%. This value indicates that there is a highly positive awareness of BCI games among researchers.

On the other hand, 61% of users and 39% of developers agreed that BCI was now on the market or would be available within 6 years. This leads us to wonder what the market situation really is. To gain some idea of the state of BCI games on the market, we investigated BCI games through mobile application stores, such as the “app stores” of two companies: Google (www.google.com) and Apple (www.apple.com). However, we found few BCI-related applications; instead, the search yielded irrelevant applications with names that included “brain” or “meditation”. On the other hand, we found that EEG device companies often support or sell their applications and some have their own application stores. Representative companies are Emotiv (www.emotiv.com) and Neurosky (store.neurosky.com). These companies already occupy large sectors of the market and their products are well-recognized among researchers, as well as by the general public. They provide various applications, such as utilities, games and software for biofeedback and well-organized developer kits are also supported to facilitate development of customized applications. This trend for companies to sell both hardware and software is anticipated to continue for some time due to two characteristics of BCI:

- First, the existing device requires a terminal to receive and process the brain signals and run the application; in addition, BCI is not as widespread as are cell-phones.
- Second, the BCI market has not matured sufficiently to expect the voluntary contribution of the third parties that develop applications, as we observed in the success of Apple's mobile devices. This may be due to the absence of a standard platform.

In a report by Gartner (www.gartner.com), a market research organization, BCI was predicted to appear on the stage of “technology triggers,” where a potential technology breakthrough initiates entry into the market. The time required for BCI to reach this plateau was expected to be more than ten years. Looking at the existing commercial products, companies are in the process of trying to find a valuable item that is both usable and attractive to consumers. However, it is difficult to specify the widespread popularity of any product. Meanwhile, the media continues to provoke curiosity and interest on the part of the public by reporting futuristic stories about BCI. According to Gartner's Hype Cycle, this increasing expectation about BCI will peak and then drop, as we know that the current technological status of BCI is limited in its ability to satisfy the public's expectations in the near future. Therefore, with the current limitations of BCI technology, how well this interface is incorporated into games or applications is the question that researchers and developers must explore. Although much of this sounds somewhat pessimistic, there has recently been some good news on the subject. Microsoft (Microsoft Research: Computational user experiences, brain-computer interfaces group) has shown an interest in BCI research and Samsung (Article “Samsung Demos a Tablet Controlled by Your Brain.” www.technologyreview.com/news/513861/samsung-demos-a-tablet-controlled-by-your-brain) demonstrated that its tablet product could be controlled by BCI. The actions of these major companies likely will boost enhancements of BCI technology.

### 5.4. Towards the Advent of BCI Games

Current technology in BCI still has many problems, such as long training time, low reliability and performance, inconvenient hardware, poor signal quality, and so on. Researchers have investigated solutions to these frequently mentioned obstacles. Thanks to their efforts, we have seen advances that may bring widespread use of BCI to the fore. Hardware companies have released both inexpensive and sophisticated EEG headsets [18] and researchers have reported the development of new signal processing algorithms [27,42] that reduce calibration time [43–46] and improve accuracy [47–51]. Looking at enhancements in performance, for example, a BCI spelling system was able to type about 0.5 characters per minute in 1999 [52], while ten years later a German group demonstrated performance exceeding 7 characters per minute [53]. Now, after just 10 min of training, it is possible to type up to 10 characters per minute with the commercial products available (www.intendix.com). Despite these achievements, however, one may ask the question, “*Can we play games with that application?*” Compared to conventional interfaces, such as keyboards, on which people normally can type several hundred characters per minute, BCI appears to be less attractive. However, BCI is quite promising in providing a new channel of communication for people. For example, if movement related brain signal would be detected before the actual movement, BCI may achieve faster and more private communication than the conventional interfaces requiring the actual movement [14,54]. Although it sounds implausible now, the potential market for healthy people is expected to be high and publicity about the technology is growing. In fact, it was reported that healthy people are the major group (more than one million persons) that will use commercial BCI technology, primarily for gaming [16]. Moreover, this is the right time for the public to accept a new interface. The touch screen has been incorporated already into smart phones and people play games with natural and intuitive body movements. Further, voice recognition phone sales are expected to reach 1 billion units (www.strategyanalytics.com). Given these trends, we need to consider how BCI can enter mainstream markets, even though it still has certain limitations. We specify here three crucial elements that may help BCI games lead market based expansion.

First, standards should be established as soon as possible. As we observed in the responses from game developers, the technical standard seems to be critical in the development of BCI games. Moreover, current BCI hardware and software are making the transition from isolated demonstrations to commercial development [55]. In order to encourage BCI game development, hardware and software at least must be standardized. In research, there have been such trials and advances in software concepts. “BCI2000” [56] and “OpenVibe” [57] facilitate BCI research by providing researchers with the ability to integrate different acquisition hardware, customized algorithm codes, and even applications easily and simultaneously. However, these platforms are for research use only, and therefore, it may be difficult for game developers to work with them as they do not have the same expertise. The need to implement platforms that are easier for game developers to use should be a primary concern within the BCI community [58]. On the other hand, hardware compatibility is also important, even though it may be less critical than software. An initial step in this process has been made; “OpenBCI” (www.openbci.com) is compatible with any type of electrode and can interface with Arduino, an open-source electronic prototyping hardware platform that allows the creation of interactive electronic objects. Apart from these technical points, other standards, such as agreement on file formats, ethical procedures, media reporting guidelines, terms and definitions, and benchmarks for the comparison of different systems [16] will probably influence the acceptance of BCI and BCI games.

Second, gameplay is a critical consideration in the development of BCI games. In our survey, one game developer (not researcher) indicated the significant influence of gameplay with an example: “*A game is likely to fail if it does not have [an] interesting story or unique game system even though the technology in [the] game device looks fancy. For example, ‘Doom3’ was the greatest in the notable graphic aspects, but finally ‘Half-life’ succeeded (‘Doom’ and ‘Half-life’ are games centered on gun and projectile weapon-based combat; these are played through a first-person perspective. Therefore, it is called a first-person shooter (FPS) game. “Half-life” proved to be revolutionary in a number of ways in the history of FPS games. It featured stunning graphics, a highly immersive game story, and gameplay. For a brief history of FPS, please refer to “History of First Person Shooter (FPS) Games.” (www.freeinfosociety.com/article.php?id=128)). ‘Gameplay’ is the one that jumps over the technological halo effect*”. In addition, there are several recent reports to emphasize gameplay in the similar context. Ko *et al.* [59] reviewed gameplay in existing BCI games and demonstrated their efficiency with respect to the existence of BCI in game playing. Gürkök *et al.* [20] tried to construct a BCI game framework from their research experiences in both the BCI and games communities. They indicated that “challenge,” “fantasy” and “sociality” made a difference in BCI games, increasing users' motivation to play. BCI depends on what control paradigm is used, so the method of interaction may vary. This means that game developers and designers should understand the characteristics of a specific BCI in order to place its function in an adequate position for interaction. For instance, training procedures may be conducted while the system quantifies the user's distribution of attention at the beginning of playing a slot car racing game [8] or a designer may put an SSVEP component on the enemy's heartbeat, thereby allowing a gamer to know intuitively the vital point at which it can be killed [33]. In this sense, one study investigated gameplay in this genre thoroughly and provided guidelines for the use of a specific BCI [21]. These studies suggested that it is possible to develop an enjoyable game and prevent the production of a substandard game where simply connecting with the BCI at all is difficult. What are needed now are guidelines or well-organized items that will enable us to evaluate the appropriate combination of a particular BCI and a game. In the game field, an exemplary study was conducted [60] and heuristics such as the game interface, mechanics, gameplay and narrative were integrated into a validated model. The model for evaluating a player's enjoyment of BCI games consisted of eight elements: concentration; challenge; skills; control; clear goals; feedback; immersion, and social interaction. Because these are the common elements for the evaluation of enjoyment, it is expected that they will be helpful in proposing development guidelines and evaluation criteria for effective game play in BCI games.

Third, the integration may constitute a breakthrough in overcoming the limitations of BCI. Given the low reliability and performance of BCI compared to conventional interfaces, the combination with other signals or systems is the inevitable option required to allow BCI to be used in game playing [61,62]. In fact, for the purposes of control, BCI does not seem to be a good choice; instead, BCI may be used as a secondary input. This issue was discussed in Section 5.2. However, it may be possible to use BCI if we apply its outcomes probabilistically for cross checking or improving overall accuracy. This integration can be accomplished with other biosignals between different BCIs referred to as hybrids [63,64], and with other interfaces [59]. For multimodal information integration, the basic framework for data, features, and decision levels has been conceptualized [24]. System integration is also important; this is related to the low functionality of BCI. What BCI does is to provide information extracted from brain signals. This means that BCI cannot be used as a stand-alone system. To make it applicable, integration with existing systems or game devices is needed. Simply put, BCI can be connected to a smart phone. However, we may expand the application area further by constructing a well-devised BCI interface. For example, it may be possible to use BCI as a plug-and-play interface for Microsoft “Xbox” or Sony “PlayStation” (www.playstation.com). Perhaps this direction is more promising than developing BCI as its own market. Interestingly, one company continues development efforts to integrate BCI into entertainment content. They have produced BCI entertainment ideas and products: “Necomimi” (moving cat's ear), “Shippo” (moving tail), and “Neurocam” (recording videos of interesting scenarios: www.neurowear.com).

### 5.5. Limitations and Considerations in the Survey

In this section, we discuss certain implicit and explicit limitations of our survey. First, the participant sample is somewhat biased. Among the 294 participants in this online survey, most were from Asia, North America and Europe. Therefore, it cannot reasonably reflect the opinions of people from other continents. Recruitment of participants was limited because it was conducted only online, its promotion was campaigned primarily online, and the questionnaire was accessible only through the internet. Thus, this survey did not cover groups that are not active in social network services or involved in games. Particularly, it would be very interesting to investigate thoroughly the opinions of patients (clinical users) who have tried BCI technology. This will be addressed in future work.

Second, the questionnaire did not address certain sophisticated issues. First, we did not consider the possibility of multiple roles of participants. For example, one can be both a researcher and a gamer. A researcher who also enjoys games may have different ideas or opinions compared to one who does not play games. Such a point may influence some findings from this survey. Considering the increase in BCI research [65–67] on user experience (UX), the questions related to UX would be quite interesting, as they would provide information about what is more familiar, acceptable and even intuitive. Further, we found that a few questions may have led respondents implicitly to more positive opinions. For example, we observed that users gave the lowest number of points to “Lie detector”, with only 45% positive opinions. Considering these issues, one should use care in drawing conclusions from this study.

Third, this survey was conducted only once. As a result, it may be difficult to draw sound and statistically significant conclusions. To achieve such confident global assessments of BCI games, repeated surveys or administration of surveys both on- and offline would be greatly beneficial. However, conducting such comprehensive surveys of large samples would be too costly and would require a large research collaboration network. Due to such difficulties, this survey was conducted once and only online. With this limitation, the Wilcoxon rank-sum test was conducted after digitization of opinions. Nevertheless, we believe that this study is a meaningful step in assessing the interactions among stakeholders in BCI games.

## 6. Conclusions

In the past few years, the potential of BCI for healthy users has attracted considerable attention. Among various applications, entertainment and games are expected to constitute a large market of potential users. In the role of producers, researchers and game developers play different roles, while users are the undeniable consumers. The feedback we obtained from these stakeholders with regard to BCI games is important for the development of high quality games. For this purpose, we presented and summarized their opinions from a survey. Our results confirmed that both the same and different opinions existed among the three groups. This means that further opinion exchange and continuing feedback among these groups are essential. Researchers must think about BCI from the viewpoint of implementers and consumers, while developers and users must remain attentive to BCI development and continue to offer their feedback. We also summarized the results of game demonstration to compare users' evaluations of passive and active paradigms, and presented briefly the state of the market. Three critical elements—standards, gameplay and appropriate integration—were discussed. Investigation of these is the direction that the BCI community must take now in order to advance BCI game market expansion. We hope that this effort will be helpful to researchers, as well as developers, and will stimulate the collaboration or exchange of opinion between those two or even all three stakeholders.

## Figures and Tables

**Table 1.** EEG Devices and their specifications. $: <200, $$: 200∼300, $$$: >;300 (unit: US Dollars).

**Device Name**	**Price**	**Year**	**Communication**	**Number of Channels**	**Sampling Rate**
Neurosky Mindset (www.neurosky.com)	$	2007	wireless	1	512 Hz
OCZ Neural Impulse Actuator (www.ocz.com)	$	2008	wired	3	1 kHz
Emotiv EPOC (www.emotiv.com)	$$	2009	wireless	14	128 Hz
PLX XWave Sonic (www.plxdevices.com)	$	2011	wireless	1	512 Hz
Neurosky Mindwave (www.neurosky.com)	$	2011	wireless	4	512 Hz
MyndPlay BrainBandXL EEG Headset (www.myndplay.com)	$$$	2012	wireless	1	512 Hz
InteraXon Muse (www.choosemuse.com)	$$	2014	wireless	4	512 Hz
Melon EEG headband (www.thinkmelon.com)	$	2014 (expected)	wireless	3	512 Hz
Emotiv Insight (www.emotiv.com)	$$ (expected)	2014 (expected)	wireless	5	128 Hz

**Table 2.** Profile records. A total of 294 respondents participated in the survey.

**Question**	**Answer**	**The Number of Respondents**	

		**Count**	**Percentage (%; N = 294)**
What is your gender?	Male	208	71
	Female	86	29

How old are you?	Under 10 years old	0	0
	Between 10 and 19 years old	16	5.5
	Between 20 and 29 years old	183	62
	Between 30 and 39 years old	71	24
	Between 40 and 49 years old	16	5.5
	Over 50 years old	8	3

Where do you live primarily?	Asia	161	55
	South America	10	3
	North America	64	22
	Europe	53	18
	Africa	2	1
	Australia (Oceania)	4	1

How many hours a day do you spend playing games (including computer games, mobile games, and console games)?	I do not play games	57	19
	Less than 30 min	95	32
	30 min∼1 h	43	15
	1∼2 h	50	17
	More than 2 h	49	17

If employed, in what sector are you employed?	Researcher	90	31
	Game developer	36	12
	Neither (User)	168	57

## Appendix

### A.1. Questionnaire

The questionnaire used for the online survey can be found in the supplementary materials. The form consisted of four sections: “Introduction”, “Background”, “Brief Introduction about Brain-Computer Interface technology” and “Questions”. The first three sections were given to all participants in the same format, while the last section (“Questions”) was asked in a different manner according to the group surveyed (users, developers or researchers). Identifier ([U], [D], [R]) on each question indicates to which group it was given.

### A.2. BCI Game Demonstration

BCI game demonstration by our research team at the HCI of Korea 2012 and Korea Computer Congress 2012 is described in detail in the appendix. We presented the games that were developed, how gamers' feedback was evaluated, and evaluation results.
